# Supplementary material for: MetaRibo-Seq measures translation in microbiomes
Source: Nat Commun. 2020 Jun 29;11:3268. doi: 10.1038/s41467-020-17081-z (PMC7324362; doi:10.1038/s41467-020-17081-z)
Supplement: Supplementary file 10 — Supplementary Data 7 [file 41467_2020_17081_MOESM10_ESM.zip › File2/Confidence_High_Taxonomy/104625_out.krona.html]

Javascript must be enabled to view this page.

members
magnitude
magnitudeUnassigned
count
unassigned
taxon
rank

104625\_out

3

3
2
superkingdom

3
976
phylum

200643
class
3

order
171549
3

1
family
815

816
genus
1


SRS077589\_contig\_number\_404
species
46506
1

family
1853231
1

1
283168
genus

SRS148874\_contig\_number\_7431

1
family
171550

1
239759
genus

1

SRS045826\_contig\_number\_21984
species
214856
